# The development of murine bone marrow-derived mast cells expressing functional human MRGPRX2 for *ex vivo* and *in vivo* studies

**DOI:** 10.3389/fimmu.2024.1523393

**Published:** 2024-12-19

**Authors:** Maram Bawazir, Saptarshi Roy, Hydar Ali

**Affiliations:** ^1^ Department of Basic and Translational Sciences, School of Dental Medicine, University of Pennsylvania, Philadelphia, PA, United States; ^2^ Department of Oral Diagnostic Sciences, Faculty of Dentistry, King Abdulaziz University, Jeddah, Saudi Arabia

**Keywords:** mast cells, MRGPRX2, LL-37, substance P, retrovirus, MrgprB2

## Abstract

**Introduction:**

A subtype of human mast cells (MCs) found in the skin and to a lesser extent in the lung and gut express a novel G protein-coupled receptor (GPCR) known as Mas-related GPCR-X2 (MRGPRX2, mouse counterpart MrgprB2). In addition to drug-induced pseudoallergy and cutaneous disorders, MrgprB2 contributes to ulcerative colitis, IgE-mediated lung inflammation and systemic anaphylaxis. Interestingly, most agonists activate MRGPRX2 with higher potency than MrgprB2. In this study, we sought to replace mouse MrgprB2 with human MRGPRX2 and to study receptor function *ex vivo* and *in vivo*.

**Methods:**

MrgprB2^−/−^ bone marrow (BM) cells were transduced with retrovirus encoding MRGPRX2 and differentiated into BMMCs (MRGRPX2-BMMCs) *ex vivo*. Cell surface MRGPRX2 expression was determined by flow cytometry. Effects of substance P (SP) and LL-37 on Ca^2+^ mobilization, degranulation and TNF-α generation were determined. MRGPRX2-BMMCs were engrafted intraperitoneally into MC-deficient W^sh^/W^sh^ mice. After 6–8 weeks, immunofluorescence staining was performed on peritoneal lavage cells (PLCs), and sections of small intestine and colon with anti c-Kit and anti-MRGPRX2 antibodies. SP-induced degranulation in PLCs obtained from engrafted mice was determined.

**Results:**

MRGPRX2-BMMCs expressed cell surface MRGPRX2 and responded to both SP and LL-37 for Ca^2+^ mobilization, degranulation and TNF-α generation. Furthermore, W^sh^/W^sh^ mice engrafted with MRGPRX2-BMMCs expressed the receptor in peritoneal, intestinal and colonic MCs. In addition, PLCs from engrafted mice responded to SP for degranulation.

**Conclusion:**

Replacing mouse MrgprB2 with functional human MRGPRX2 in primary BMMCs and their engraftment in MC-deficient mice demonstrated the expression of this receptor in different tissues, which provides unique opportunities to study receptor signaling *ex vivo* and disease phenotype *in vivo.*

## Introduction

Mast cells (MCs) are tissue-resident immune cells that are present in close proximity to sensory nerve endings and blood vessels in the host environment interface, including the skin, mucosal lining of the respiratory tract, oral mucosa, and gastrointestinal tract and are best-known for their roles in IgE-(FcϵRI)-mediated hypersensitivity reactions (1, 2). Recently, it has been shown that cutaneous MCs express a novel G protein-coupled receptor (GPCR) known as Mas-related GPCR-X2 (MRGPRX2, mouse counterpart MrgprB2), which is activated by Food and Drug Administration (FDA) approved cationic drugs, epithelium-derived antimicrobial host defense peptides (HDPs) such as the cathelicidin (LL-37), chemokines and the neuropeptide substance P (SP) (3–9). The generation of MrgprB2^−/−^ mice has been instrumental in demonstrating the receptor’s involvement in drug-induced pseudoallergic reactions, neurogenic inflammation, atopic dermatitis, allergic contact dermatitis, and rosacea (3, 4, 6, 10–13).

Previous histological studies led to the characterization of MCs into two main categories: mucosal MCs (MMCs) and connective tissue–type MCs (CTMCs) (14). MMCs are found mostly in the mucosal lining of the gut and lungs, and CTMCs are found in the skin and peritoneal cavity (15). More recently, using 10X scRNA-seq transcriptional profiles, Tauber et al. (16), identified two mouse MC populations based on the expression of MrgprB2, with a large proportion of skin and peritoneal cavity CTMCs being MrgprB2 positive and gut MMCs being MrgprB2 negative (16). Using MrgprB2-reporter mice, the authors demonstrated that MrgprB2-expressing CTMCs are not restricted to the skin and peritoneal cavity but are also present in the lung, gut, heart, skeletal muscle, and uterus. Systemic anaphylaxis reactions such as food allergy or drug allergy are potentially life-threatening allergic reactions for which pathological features are often observed in many tissues, including the gastrointestinal tract, skin, lungs, and cardiovascular system. Interestingly, the deletion of MrgprB2^+^-CTMCs protects mice from peanut-induced food allergy and IgE-mediated systemic anaphylaxis (16). Moreover, we recently showed that MrgprB2^–/–^ mice are protected from IgE-mediated systemic anaphylaxis and Type 2 lung inflammation (17, 18). In addition, Van Remoortel et al. (19), showed that MrgprB2-dependent MC activation plays a crucial role in acute colitis. It is noteworthy that while early studies indicated that MRGPRX2 is highly expressed in human cutaneous MCs, it is now realized that lung and colonic MCs express the receptor and may participate in asthma and ulcerative colitis (20–24).

Although the same set of cationic ligands activates both human MRGPRX2 and mouse MrgprB2, the EC_50_ values (concentration required to give 50% response) are generally higher for the mouse when compared to the human receptor (3). This difference is attributed to the low amino acid sequence identity (~53%) between MrgprB2 and MRGPRX2 (25). Moreover, we have recently shown that a specific small molecule MRGPRX2 inverse agonist inhibits degranulation in response to a variety of cationic ligands in human MCs without affecting responses to the same agonists in mouse MCs (26). The presence of these species-specific differences poses a significant challenge to delineate the underlying mechanisms of MRGPRX2-mediated diseases and the development of targeted therapy in mouse models. Three groups, including our lab, recently reported the development of transgenic mice in which the endogenous MrgprB2 was replaced with a functional MRGPRX2 (MRGPRX2-KI mice) (27–29). Thus, MRGPRX2-KI mice provide a useful preclinical model to study the role of MRGPRX2 in MC-mediated inflammatory disorders. Recent studies led to the identification of several gain and loss of function missense variants of MRGPRX2 (30–32). In addition, distinct binding pockets on MRGPRX2 have been identified, and the receptor has been shown to couple to different G proteins (33, 34). Therefore, additional models are required that would facilitate the study of how MRGPRX2 modification and its downstream signaling regulates receptor function *ex vivo* and disease phenotype *in vivo.*

Retrovirus-mediated gene transfer is a powerful tool that can be used to understand gene functions in primary cells (35). This system has been used to study how modification of FcϵRI and its signaling regulates the functions of mouse BMMCs *ex vivo* (36, 37). In the present study, we used a retrovirus to express functional MRGPRX2 into MrgprB2^−/−^ bone marrow derived MCs (MRGPRX2-BMMCs) to study receptor regulation in primary MCs. Engraftment of MC-deficient W^sh^/W^sh^ mice with BMMCs cultured from genetically modified mice have been extensively used to study the role of MC-specific gene modifications in host defense, allergic asthma and cutaneous disorders (38–42). We also engrafted MRGPRX2-BMMCs into W^sh^/W^sh^ mice to determine tissue distribution and to assess their functional properties. Our ability to express human MRGPRX2 in mouse BMMCs and to engraft these cells into W^sh^/W^sh^ mice with retention of functional receptor provides a unique opportunity to delineate how structural modification of the receptor and its signaling modulates receptor function *in vivo.*

## Material and methods

### Reagents

Most of the cell culture reagents, including DNP-specific mouse IgE were purchased from Invitrogen (Carlsbad, CA, USA). LL-37 was obtained from GenicBio Limited (China), and Substance P (SP) were purchased from AnaSpec (Fremont, CA, USA). Recombinant mouse interleukin-3 (IL-3) was obtained from Peprotech (Rocky Hill, NJ, USA). DAPI was purchased from Molecular Probes (Eugene, OR, USA). DNP-BSA and P-nitrophenyl-N-acetyl-β-D-glucosamine (PNAG) were obtained from Sigma-Aldrich (St. Louis, MO, USA). Fura-2 acetoxymethyl ester (Fura-2 AM) was obtained from Abcam (Cambridge, MA, USA). Lipofectamine™ 2000 transfection reagent was purchased from Invitrogen (Carlsbad, CA, USA). PE-conjugated anti-human MRGPRX2 (Clone K125H4, Catalog 359004), APC-conjugated anti-mouse c-Kit (Clone 2b8, Catalog 105812), and APC-conjugated anti-mouse CD107a (Clone 1D4B, Catalog 121614) antibodies were obtained from BioLegend (San Diego, CA, USA). FITC-conjugated anti-mouse FcϵRI (Clone MAR-1, Catalog 11-5898-85) was obtained from eBioscience™. Retro-X™ concentrator was purchased from Takara Bio Inc. (Japan). Plasmid encoding pMXs-puro vector was obtained from (Addgene #74203) (43).

### Mice

W^sh^/W^sh^ and C57BL/6 mice were purchased from the Jackson Laboratory (Bar Harbor, ME, USA). MrgprB2^−/−^ mice were generated as previously described (30). Mice were housed in pathogen-free conditions and autoclaved hardwood bedding. Female mice (ages 4 to 6 weeks) were used for experiments. All experiments were approved by the Institutional Animal Care and Use Committee at the University of Pennsylvania.

### Culturing Plat-E cells and virus particles generation

Plat-E cells (1.5x10^6^) were plated in a 60-mm tissue culture plate, resuspended in 5 ml of Dulbecco’s modified Eagle’s medium (DMEM) antibiotic/supplements-free and incubated overnight at 37°C in a humidified 5% CO_2_ incubator. The next day, medium was removed and transient transfection was carried out with 8 μg of pMX-puro encoding MRGPRX2 or empty vector using Lipofectamine™ 2000 DNA transfection reagent, according to the manufacturer protocol (32, 44). Cells were then incubated in an antibiotic-free medium for 48 h at 37°C with 5% CO_2_. Supernatants were collected and filtered through a 0.45 µm filter and virus particles were incubated with Retro-X™ concentrator (1:3) (Takara Bio Inc, Japan) for 4-6 h at 4°C. Next, cells were centrifuged at 1,500×*g* for 45 min at 4°C, supernatants were removed, pellet were resuspended in 500 μl of Roswell Park Memorial Institute 1640 Medium (RPMI 1640) and stored immediately at -80°C till further use.

### Generation of transduced murine BMMCs

Bone marrow (BM) cells were harvested from the femurs of MrgprB2^−/−^ mice. Cell suspension was strained through a 70-mm filter mesh and incubated overnight at 37°C with 5% CO_2_. After 24 h, cell transduction was carried out with purified retrovirus particles encoding MRGPRX2 or vector-control. Briefly, purified virus-containing RPMI 1640 medium (500 μl) and polybrene (4 μg/ml; 5 ml) were added to BM cells (1x10^7^) in 4.5 ml antibiotic-free RMPI medium and mixed gently. Cells were incubated at 37°C with 5% CO_2_. After 8 h, cell suspension was collected, centrifuged at 400×*g* for 5 min, and resuspended in fresh complete RPMI 1640 medium containing 10% fetal calf serum (FCS), 5% Non-Essential Amino Acid (NEAA), β-mercaptoethanol (45.6 µM), penicillin (100 IU/ml), streptomycin (100 mg/ml), recombinant mouse IL-3 (10 ng/ml). After 24 h, puromycin (2 µg/ml) was introduced to the cell culture medium. Cells were cultured and differentiated into BMMCs in the presence of puromycin for two weeks and mIL-3 for 4 weeks. The purity of cultured cells was determined by flow cytometry using anti-mouse c-Kit and FcϵRI antibodies to reach ~95%. Cells were used within 4-8 weeks.

### Flow cytometry

BMMCs (0.5x10^6^ cells) were washed and suspended in FACS buffer (PBS containing 2% FCS and 0.02% sodium azide). To assess MC differentiation, cells were incubated with APC-conjugated anti-mouse c-Kit and FITC-conjugated anti-mouse FcϵRI antibodies in the dark for 30 min at 4°C. Cells were incubated with PE-conjugated anti-human MRGPRX2 antibody to measure cell surface receptor expression. Cells were washed in FACS buffer and fixed in 1.5% paraformaldehyde. Data was acquired using a BD LSR II flow cytometer (San Jose, CA, USA) and analyzed with the FlowJo software version 10.8.1 (Tree Star Inc., Ashland, OR) (23, 31).

### Ca^2+^ mobilization assay

Ca^2+^ mobilization was determined as described previously (26). Briefly, transduced BMMCs (0.2×10^5^) were loaded with Fura-2 acetoxymethyl ester (1 mM) in HEPES buffer containing 0.1% of bovine serum albumin (BSA) in the dark for 30 min at 37°C, followed by de-esterification for an additional 15 min at room temperature. Cells were washed and resuspended in the buffer and Ca^2+^ mobilization was measured for 600 s interval with the addition of SP or LL-37 (10 μM) at 100 s. Ca^2+^ signals were determined by measuring the fluorescence ratio between dual excitation wavelengths of 340 and 380 nm and an emission wavelength of 510 nm using the Varioskan LUX Multimode Microplate Reader (ThermoScientific, Waltham, MA, USA).

### Degranulation measured by *β*-hexosaminidase release assay

BMMCs expressing MRGPRX2 (MRGPRX2-BMMCs) or control cells (control-BMMCs) were seeded into a 96-well plate (5×10^4^ cells/well) in a total volume of 50 μl HEPES buffer containing 0.1% BSA and exposed to different concentrations of SP, LL-37 or vehicle control for 30 min at 37°C. For a subset of studies, peritoneal lavage cells (PLCs) from engrafted W^sh^/W^sh^ (1.5x10^5^ cells/well) were stimulated with SP (10 µM), C48/80 (5 µg/ml) or vehicle control for 30 min at 37°C. To determine the total *β*-hexosaminidase release, unstimulated cells were lysed in 50 μl of 0.1% Triton X-100. Aliquots (20 μl) of supernatants were incubated with 20 μl of 1 mM p-nitrophenyl-N-acetyl-b-D-glucosamine (PNAG) for 1.5 h at 37°C. Finally, 250 μl of stop solution was added (0.1 M Na_2_CO_3_/0.1 M NaHCO_3_) to stop the reaction. The absorbance was measured at a wavelength of 405 nm using a Versamax microplate spectrophotometer (Molecular Devices, San Jose, CA, USA). Percentage of *β*-hexosaminidase degranulation was calculated by dividing the *β*-hexosaminidase release in the sample by total *β*-hexosaminidase release.

### Degranulation measured by the surface expression of CD107a

MC degranulation was also assessed by flow cytometric measurement of the cell surface expression of CD107a following agonist stimulation. MRGPRX2-BMMCs or control-BMMCs (0.2x10^6^) were stimulated with SP or LL-37 (10 μM) at 37°C. After 5 min, cells were fixed with fixation buffer (Biolegend, Catalog 420801) for 15 min at room temperature. Cells were washed with FACS buffer and non-specific binding was blocked with 1% BSA in PBS for 30 min at 4°C. Cells were exposed to APC-conjugated anti-mouse CD107a for 30 min at 4°C. Cell surface expression of CD107a was acquired by a BD LSR II flow cytometer (San Jose, CA, USA) and analyzed with the FlowJo software version 10.8.1 (Tree Star Inc., Ashland, OR).

### MRGPRX2 internalization

MRGPRX2 internalization was determined as described previously (26). Briefly, MRGPRX2-BMMCs (0.3x10^6^) were treated with SP or LL-37 (10 μM) for 30 min at 37°C with untreated cells served as control. Cells were washed with FACS buffer and incubated with PE-conjugated anti-human MRGPRX2 antibody at 4°C in the dark for 30 min. Cells were then washed and fixed in 1.5% paraformaldehyde. Data was acquired using a BD LSR II flow cytometer (San Jose, CA, USA) and analyzed with the FlowJo software version 10.8.1 (Tree Star Inc., Ashland, OR).

### Enzyme-linked immunosorbent assay (ELISA)

ELISA was performed according to the manufacturer’s protocol (DuoSet ELISA kits, R&D systems) to quantify the release of murine TNF-α. Briefly, MRGPRX2-BMMCs were suspended in a complete RPMI medium and seeded in a 24-well sterile plate (0.5×10^6^ cells/well) in the absence or presence of SP or LL-37 (10 µM) for 16 h with untreated cells served as control. Cells were centrifuged and supernatants were collected to measure TNF-α release (45).

### Engraftment of BMMCs into mast cell-deficient W^sh^/W^sh^ mice

Engraftment of transduced BMMCs was carried out as previously described (46). Briefly, MRGPRX2-BMMCs or control-BMMCs (2x10^6^) were resuspended in cold DMEM (200 μl), kept on ice and injected intraperitoneally (i.p.) into 4-6 weeks old recipient MC-deficient W^sh^/W^sh^ mice. Evaluation of the engraftment efficacy and the *in vivo* experiments were carried out 6-8 weeks post-engraftment (46, 47).

### Peritoneal lavage cells isolation

Engrafted W^sh^/W^sh^ mice were euthanized, and peritoneal lavage cells (PLCs) were collected by flushing the peritoneal cavity with sterile cold PBS (10 ml). Cells were centrifuged at 400×*g* for 10 min at 4°C, resuspended in HEPES buffer, and utilized for *β*-hexosaminidase release assay, as described above.

### Mast cell staining

PLCs collected from the engrafted mice were washed and resuspended in PBS. Cell suspension (1x10^5^) was added to cytofunnel attached to microscope slide and cytocentrifuged. Slides were dried and stained with alcian/safranin or toluidine blue, as described previously (17, 48, 49). Images were acquired and visualized on a Nikon Eclipse Ni microscope. To assess degranulation at a single cell level, PLCs (0.2x10^6^) were incubated in the absence or presence of SP (10 µM, for 30 min) at 37°C, then fixed with fixation buffer. Degranulated MCs were identified by the presence of diffusely exocytosed granules following toluidine blue staining, as described previously (50–52).

### Immunofluorescence staining

Immunofluorescence experiments were performed using tissue samples embedded in paraffin. Tissue sections were deparaffinized, hydrated, antigen-retrieved, blocked with 1% BSA in PBS-T (PBS containing 0.2% Triton X-100) for 1 h in a humidified chamber, and incubated with FITC-conjugated anti-mouse c-Kit (1:500) and PE-conjugated anti-human MRGPRX2 (1:250) antibodies overnight at 4°C. Tissue sections were then washed with PBS, dried and mounted with DAPI. Images were acquired and visualized on a Nikon Eclipse Ni microscope.

### Statistical analysis

GraphPad PRISM software version 9.0.1 (San Diego, CSA) was used for statistical analysis. Results were expressed as mean ± standard error of the mean (SEM) values. SEM values were derived from at least three independent experiments. Statistical significance was measured by *t*-test, one-way analysis of variance (ANOVA) or two-way ANOVA for multiple comparisons. A *P*-value ≤ 0.05 was considered to be significant.

## Results

### Generation and characterization of MRGPRX2 expressed in murine BMMCs

**Figure 1:**
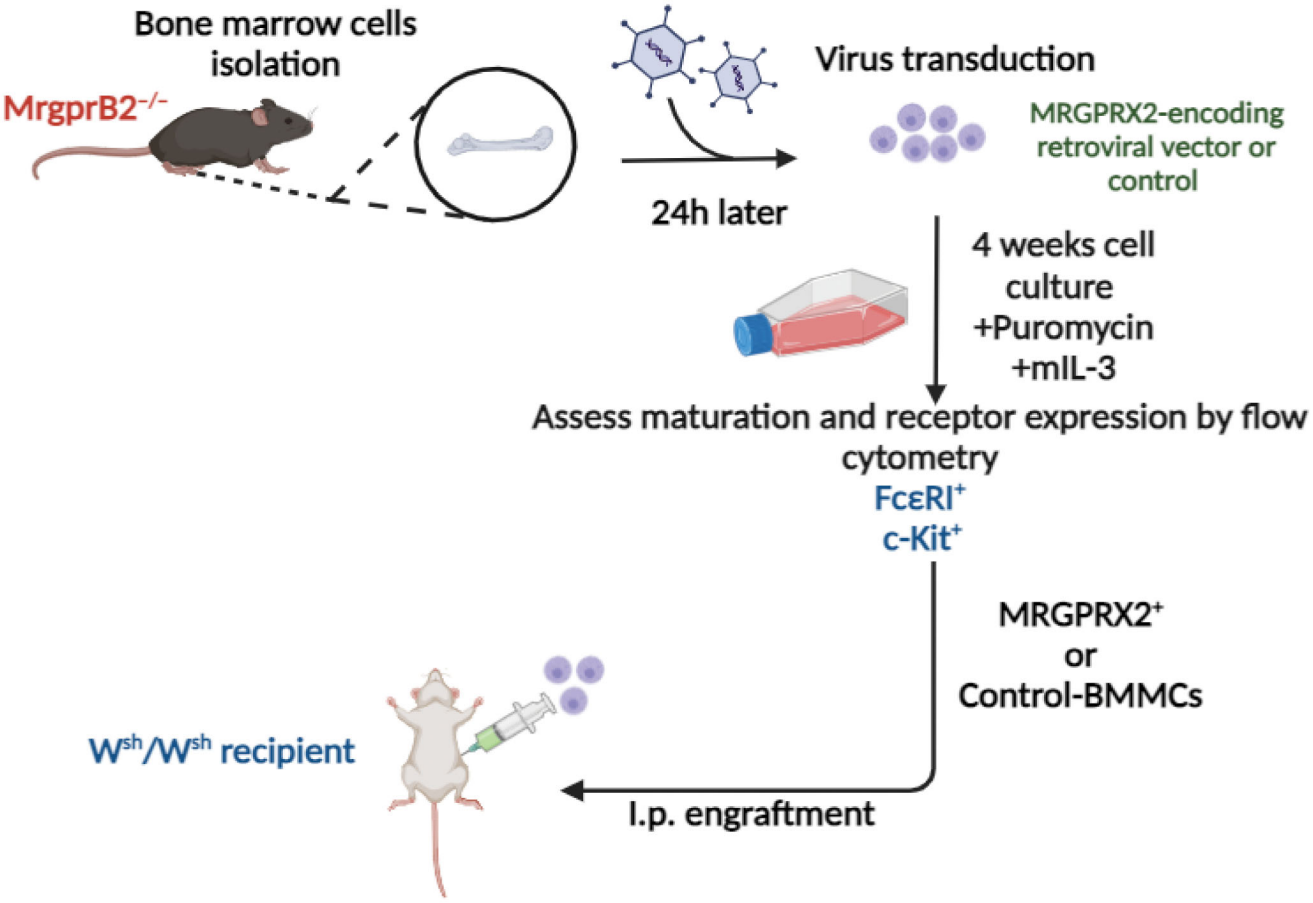
Schematics for the replacement of MrgprB2 with MRGPRX2 in mouse BMMCs, their engraftment into W^sh^/W^sh^ mice and analysis of tissue distribution *in vivo*. Bone marrow cells obtained from femurs of MrgprB2^–/–^ mice were transduced with an MRGPRX2-encoding retroviral vector or control vector and cultured in a medium supplemented with mouse IL-3 (10 ng/ml) for 4 weeks in the presence of puromycin for the first two weeks to obtain BMMCs. Expression of FcϵRI, c-Kit and MRGPRX2 were evaluated by flow cytometry and cells were used for functional studies *ex vivo*. Transduced BMMCs were also engrafted intraperitoneally (i.p.) into MC-deficient W^sh^/W^sh^ mice. These mice were used for experiments 6-8 weeks later. Created with BioRender.com.

**Figure 2:**
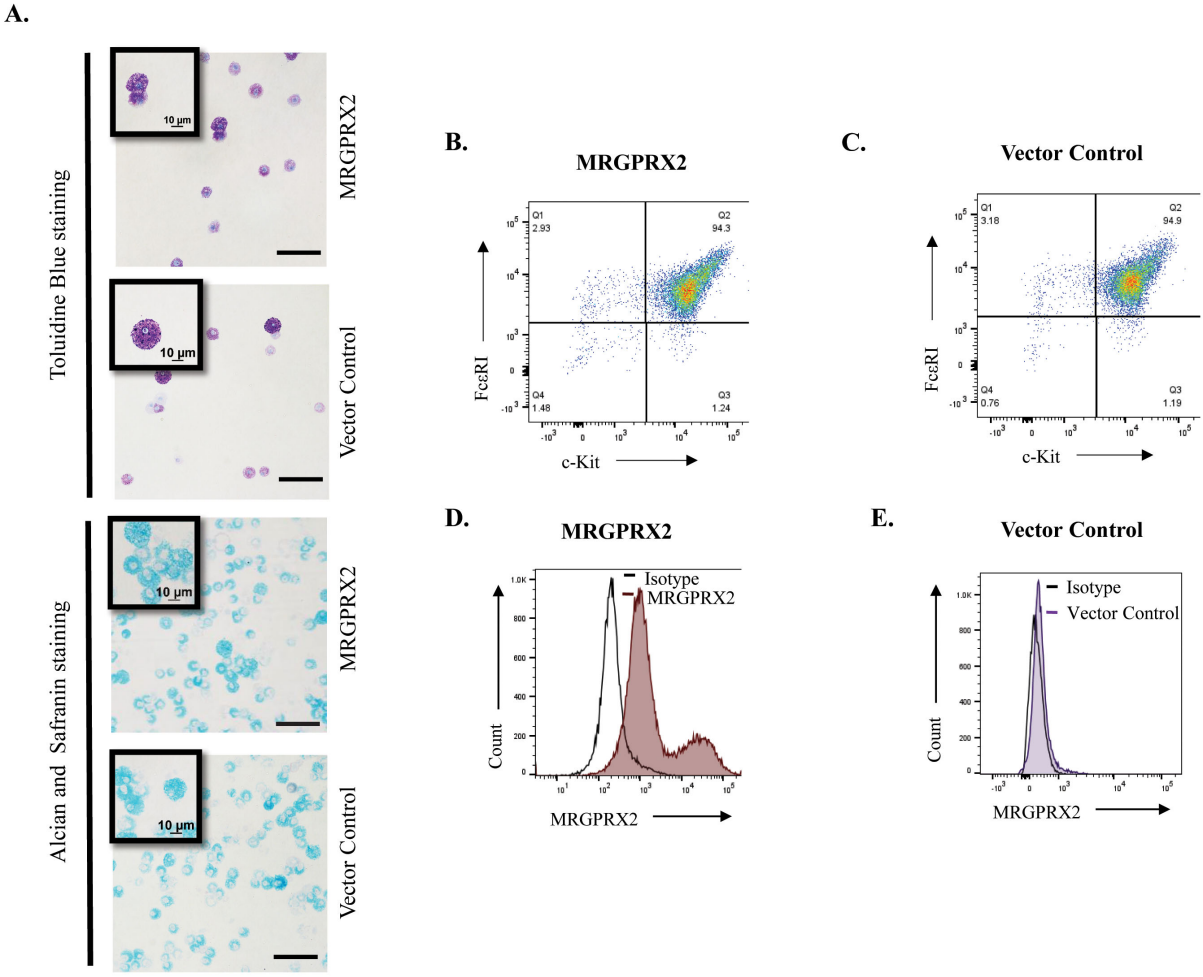
Properties of transduced BMMCs. **(A)** BMMCs stably expressing MRGPRX2, or vector control cells were cytospun, dried and stained with toluidine blue or alcian/safranin and representative microscopic images are shown (Bar =10 and 100 µm) (*n* = 3). Cells were incubated with fluorescent anti-MRGPRX2, anti c-Kit and anti-FcϵRI antibodies, and cell surface expression was determined by flow cytometry. **(B, C)** Representative flow cytometric analyses of c-Kit and FcϵRI expression on the surface of BMMCs, 4 weeks post-transduction. **(D, E)** Representative flow cytometry histograms for MRGPRX2 cell surface expression and isotype in MRGPRX2 or control BMMCs (*n* = 5).

A Moloney murine leukemia virus-based vector, pMXs-puro, has been used to stably express signaling components in murine BMMCs (53, 54). For this study, we cloned cDNA encoding MRGPRX2 into pMXs-puro plasmid and generated virus particles. BM cells from MrgprB2^−/−^ mice were transduced with a virus encoding MRGPRX2 or vector control, and selection with puromycin was initiated. Transduced cells were cultured in the presence of mIL-3 for 4 weeks for their differentiation into BMMCs (**Figure 1**). Metachromatic staining with toluidine blue marks MC granules in purple color, while alcian/safranin is commonly used to differentiate CTMCs (pink) from mucosal MCs (blue). Safranin binds to highly sulfated glycosaminoglycans like heparin, characteristic of CTMCs, while alcian blue stains the granules of mucosal MC, which contain poorly sulfated glycosaminoglycans such as heparin precursors and chondroitin sulfate E (55, 56). We found that introduction of the retrovirus vector or vector plus MRGPRX2 into BM cells and their differentiation into BMMCs did not induce a visible change in the cells’ phenotypic/morphological characteristics. BMMCs stained purple with toluidine blue and retained their immature cell linage as mucosal type MC (alcian blue^+^/safranin^-^) (**Figure 2A**). Next, we performed flow cytometry using anti-FcϵRI and anti c-Kit antibodies to show that ~95% of cultured transduced cells differentiated into MCs in the presence of IL-3 supplementation (**Figures 2B, C**). To assess cell surface expression of MRGPRX2, we used an anti-human MRGPRX2 antibody for flow cytometric studies. As shown in **Figures 2D, E**, cells transduced with virus particles encoding MRGPRX2 (MRGPRX2-BMMCs) expressed the receptor but at different levels, as two distinct subpopulations were observed but vector transduced BMMCs (control-BMMCs) did not express the receptor.

**Figure 3:**
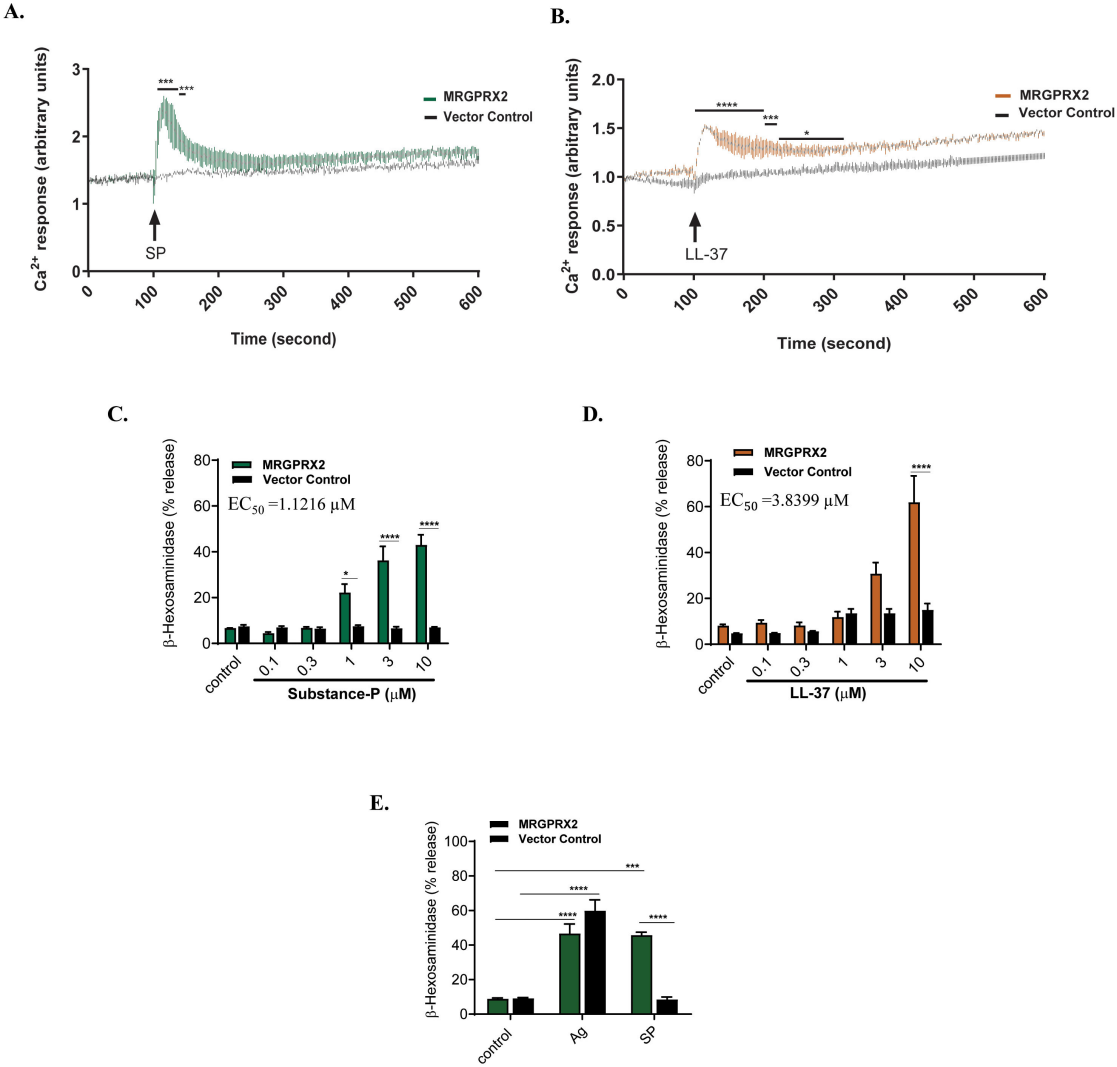
SP and LL-37 induced MRGPRX2-mediated Ca^2+^ mobilization and degranulation in BMMCs expressing MRGPRX2. BMMCs stably expressing MRGPRX2, or vector control cells were loaded with Fura-2 and time course of Ca^2+^ mobilization was determined following stimulation with **(A)** SP (3 µM) or **(B)** LL-37 (3 µM). **(C, D)** Cells were stimulated with different concentrations of SP or LL-37 and *β*-hexosaminidase release was determined. The EC_50_ values for degranulation were determined from dose-response studies, which were repeated four times utilizing a four-parameter logistic model. **(E)** Cells were primed with DNP-specific IgE (1 µg/ml, 16 h), stimulated with antigen (DNP-BSA, 30 ng/ml) or SP (10 µM) for 30 min and degranulation (*β*-hexosaminidase release) was determined. Data are expressed as mean ± SEM of at least three independent experiments. Statistical significance was determined by one-way or two-way ANOVA at a value *P< 0.05, ***P< 0.001 and ****P< 0.0001.

**Figure 4:**
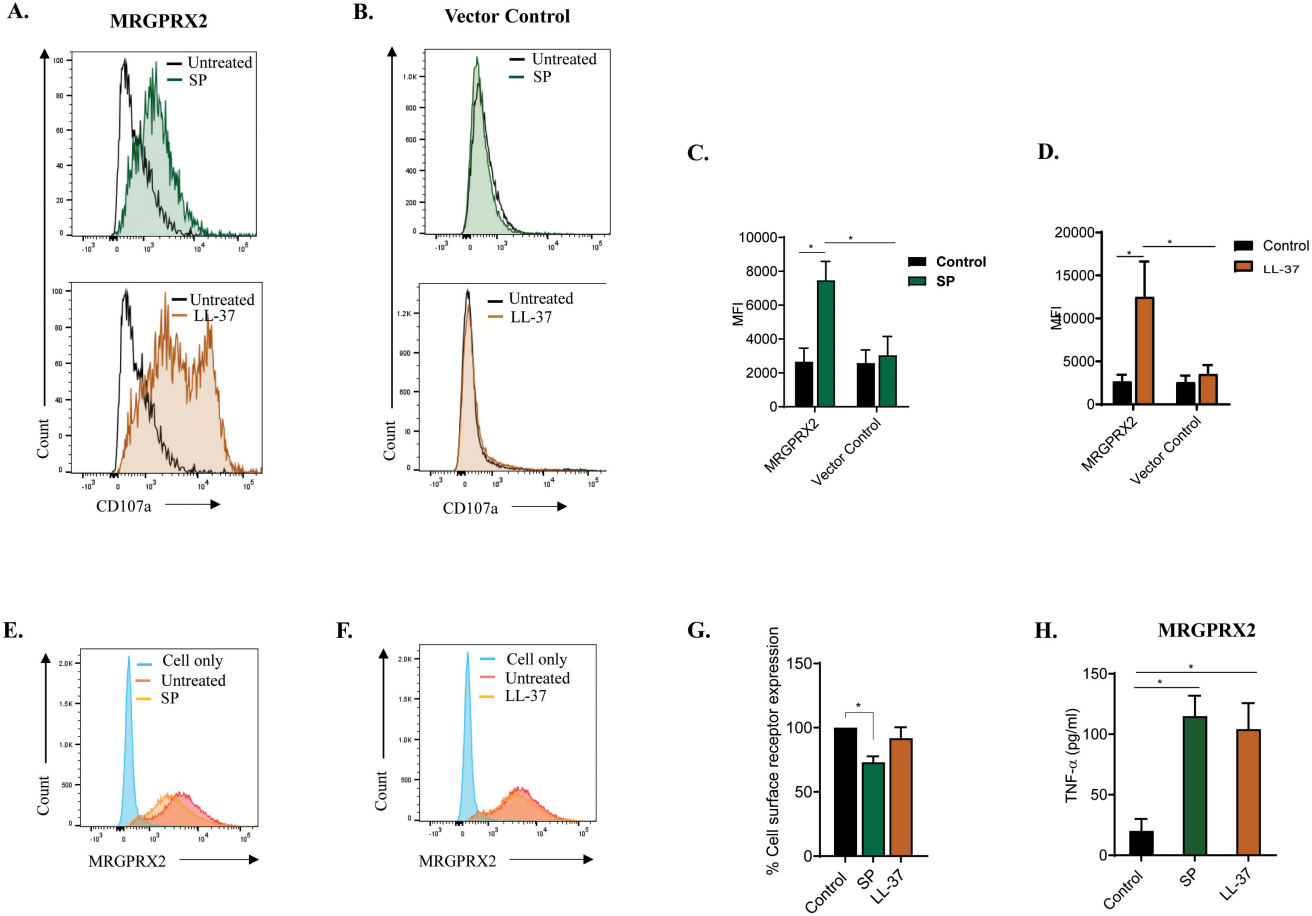
Cell surface CD107a expression, MRGPRX2 internalization and TNF-α production following stimulation with SP and LL-37. BMMCs expressing **(A)** MRGPRX2 or **(B)** vector control-BMMCs were exposed to SP or LL-37 (10 µM) for 5 min, and the surface expression of CD107a was determined by flow cytometry. Histograms are representative of at least three independent experiments. **(C, D)** The adjusted mean fluorescent intensity (MFI) level of CD107a is shown. **(E, F)** Cells were exposed to SP or LL-37 (10 µM) for 30 min and cell surface MRGPRX2 expression was determined by flow cytometry using PE-conjugated anti-MRGPRX2 antibody. **(G)** The percentage of receptor internalization was calculated using an MFI compared to the untreated controls. **(H)** Cells were incubated in the presence or absence of SP or LL-37 (10 µM) for 16 h, and the production of cytokine TNF-α was quantified by ELISA. Data are expressed as mean ± SEM. Statistical significance was determined by one-way ANOVA or two-way ANOVA at a value *P< 0.05.

To assess the functional integrity of the receptor, we tested the effect of two MRGPRX2 agonists, the neuropeptide SP and cathelicidin LL-37, for Ca^2+^ mobilization (6, 57, 58). For this, Fura-2-loaded cells were exposed to SP or LL-37 (3 µM) and Ca^2+^ mobilization was measured continuously for 600 s. We found that SP and LL-37 caused substantial Ca^2+^ response in MRGPRX2-BMMCs but not in control-BMMCs at all time points tested (**Figures 3A, B**). Although MRGPRX2 and MrgprB2 are activated by the same cationic peptides, there are important differences in the concentrations required to activate these receptors (3). Thus, in transfected HEK293 cells, EC_50_ values for Ca^2+^ mobilization by SP are 54.3 μM and 152 nM for MrgprB2 and MRGPRX2, respectively (3). Not surprisingly, SP at 100 µM induces small to variable degranulation in mouse peritoneal mast cells (PMCs) expressing MrgprB2 (45). We found that MRGPRX2-BMMCs showed a substantial degranulation in response to SP with EC_50_ of ~ 1 µM, and these responses were absent in control-BMMCs (**Figure 3C**). LL-37 also induced substantial degranulation in MRGPRX2-BMMCs but not in control-BMMCs (**Figure 3D**). To confirm that cultured and transduced BMMCs retained their characteristic IgE-mediated responsiveness, cells were sensitized with anti-DNP-specific IgE and degranulation (β -hexosaminidase release) was determined following antigen (DNP-BSA) stimulation. As shown in **Figure 3E**, IgE-mediated degranulation was similar regardless of MRGPRX2 status of BMMCs. CD107a is a granule-associated protein that undergoes externalization during MC degranulation (59). We found that both SP and LL-37 (10 µM) caused increased cell surface expression of CD107a in MRGPRX2-BMMCs but not control BMMCs as measured by flow cytometry (**Figures 4A, B**). Quantitative results are shown in **Figures 4C, D**.

While both SP and LL-37 couple to G-proteins for Ca^2+^ mobilization and degranulation, SP also promotes β-arrestin-mediated MRGPRX2 internalization, but LL-37 does not (57, 58). To determine receptor internalization, MRGPRX2-BMMCs were exposed to SP or LL-37 (10 µM), and loss of cell surface receptor expression was assessed by flow cytometry. Consistent with previous reports (57, 58), SP induced significant MRGPRX2 internalization, but LL-37 did not. Representative histograms of cell surface MRGPRX2 expression following stimulation with agonists for 30 min are shown in **Figures 4E, F**, and quantitative results are shown in **Figure 4G**. SP and LL-37 induce chemokine and cytokine generation in a human MC line, LAD2 cells and primary human skin-derived MCs endogenously expressing MRGPRX2 (4, 5). To determine if these agonists also cause cytokine generation in transduced BMMCs, MRGPRX2-BMMCs were exposed to SP or LL-37 (10 µM, 16 h), and the production of TNF-α was determined by ELISA. As shown in **Figure 4H**, both SP and LL-37 induced a significant release of TNF-α. These findings demonstrate that MRGPRX2, stably expressed in mouse BMMCs, displays functional properties similar to the natively expressed receptor in human MCs.

### Analysis and function of retrovirus generated MRGPRX2-BMMCs engrafted into W^sh^/W^sh^ mice

**Figure 5:**
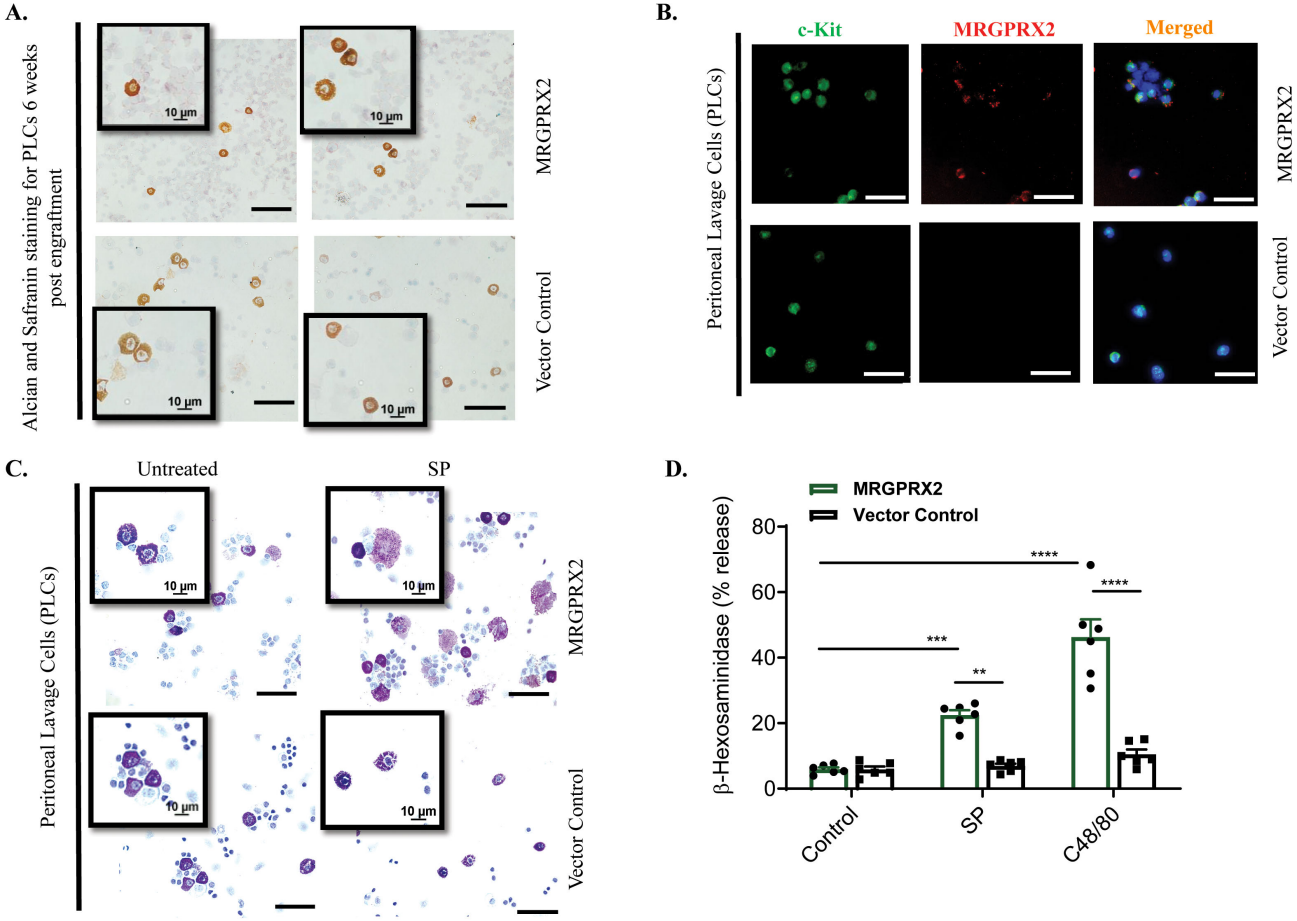
Properties of peritoneal MCs obtained from W^sh^/W^sh^ mice engrafted with transduced BMMCs. **(A)** Peritoneal lavage cells (PLCs) obtained from W^sh^/W^sh^ mice engrafted with BMMCs expressing MRGPRX2 or vector control were cytospun, dried and stained with Alcian/safranin and representative images are shown. (Bar = 50 and 10 µM). **(B)** Double immunofluorescence staining of PLCs with c-Kit (left, green), MRGPRX2 (middle, red), and merged with DAPI (right, blue). Bar = 50 μm. **(C)** PLCs were exposed to SP (10 µM) for 30 min, and degranulation was determined by toluidine blue staining. **(D)** PLCs were stimulated with SP (10 µM) or C48/80 (5 µg/ml) and *β*-hexosaminidase release was determined. Data are expressed as mean ± SEM. Statistical significance was determined by two-way ANOVA at a value **P< 0.01, ***P< 0.001 and ****P< 0.0001.

We engrafted MRGPRX2-BMMCs and control-BMMCs into the peritoneum of W^sh^/W^sh^ mice and after 6-8 weeks, PLCs were collected and stained with alcian and safranin. Before engraftment, transduced BMMCs stained blue with alcian/safranin (**Figure 2A**), but following engraftment in the peritoneum, these cells stained pink with the same dye, demonstrating maturation of MMCs to CTMCs (**Figure 5A**). To determine if the engrafted MCs in the peritoneum retained the expression of MRGPRX2, PLCs were stained with c-Kit antibody (Green; a marker of MCs) and anti-human MRGPRX2 antibody (Red). As shown in **Figure 5B**, c-Kit^+^ cells from W^sh^/W^sh^ mice engrafted with MRGPRX2-BMMCs demonstrated the expression of MRGPRX2. By contrast, c-Kit^+^ cells from mice engrafted with control-BMMCs did not express the receptor. To determine if MRGPRX2 expressed in PMCs are functionally responsive, PLCs were collected, stimulated with SP (10 µM for 30 min), fixed and stained with toluidine blue. Degranulated MCs were identified by the presence of diffusely exocytosed granules (50–52). As shown in **Figure 5C**, PLCs obtained from W^sh^/W^sh^ mice engrafted with MRGPRX2-BMMCs responded to SP for degranulation. By contrast, PLCs obtained from W^sh^/W^sh^ mice engrafted with control-BMMCs did not display diffusely exocytosed granules. We also assessed degranulation in response to SP and the classic MRGPRX2 agonist compound 48/80 (C48/80) by quantitating β -hexosaminidase release. As shown in **Figure 5D**, PLCs obtained from W^sh^/W^sh^ mice engrafted with MRGPRX2-BMMCs responded to SP and C48/80 for degranulation, but those obtained from W^sh^/W^sh^ mice engrafted with control-BMMCs did not. These findings demonstrate that MRGPRX2-BMMCs engrafted into W^sh^/W^sh^ mice differentiated into CTMCs in the peritoneum, retained cell surface receptor expression and are functionally responsive for degranulation.

**Figure 6:**
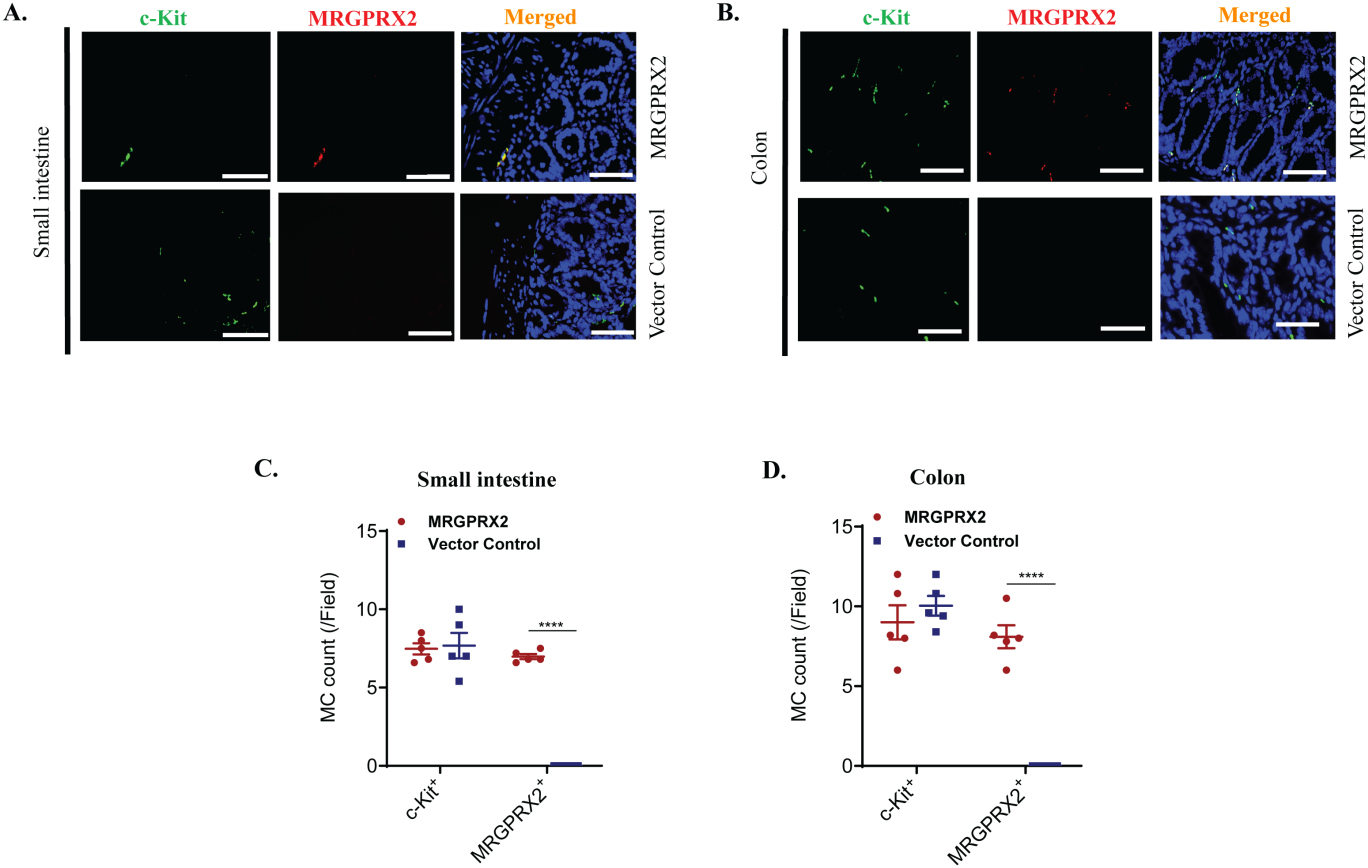
MC migration and MRGPRX2 expression post-engraftment into W^sh^/W^sh^ mice. Representative images of immunofluorescence staining of **(A)** small intestine and **(B)** colon samples obtained from W^sh^/W^sh^ mice engrafted with BMMCs expressing MRGPRX2 or vector control. Tissues were stained for c-Kit (left, green) and MRGPRX2 (middle, red) and merged with DAPI (right, blue) (*n* = 5). Bar = 50 μm. **(C, D)** Quantitative analysis of MRGPRX2 and c-Kit expression was presented as a number of MC expressing the marker per field. Data are expressed as mean ± SEM. Statistical significance was determined by a two-way ANOVA test at a value ****P< 0.0001.

BMMCs engrafted into W^sh^/W^sh^ mice via the intraperitoneal route migrate to the colon and small intestine (47). We therefore sought to determine if engrafted MRGPRX2-BMMCs migrate to these sites and if they retain the expression of the receptor. For this, formaldehyde-fixed paraffin-embedded tissues from W^sh^/W^sh^ mice engrafted with MRGPRX2-BMMCs or control-BMMCs were subjected to immunofluorescence staining with c-Kit and anti-human MRGPRX2 antibodies. Staining with c-Kit demonstrated that the introduction of MRGPRX2 has no significant impact on the number of BMMCs that migrated to the small intestine and colon (**Figures 6A, B**). Furthermore, c-Kit^+^ cells in both the small intestine and colon of W^sh^/W^sh^ mice engrafted with MRGPRX2-BMMCs express the receptor. By contrast, tissue MCs in mice engrafted with control-BMMCs did not express the receptor. Quantitative results are shown in **Figures 6C, D**.

## Discussion

The discovery that MRGPRX2 is the receptor for basic secretagogues has revolutionized the way MCs are viewed (20, 25, 60). Furthermore, the development of MrgprB2^–/–^ mice has been instrumental in delineating the role of this receptor in pseudoallergy and a variety of MC-mediated disorders (3, 6, 10, 12). It is noteworthy that although MrgprB2 is the mouse ortholog for human MRGPRX2, there are striking differences in the pharmacology of these receptors, making it difficult to translate *in vitro* studies with human MCs to the *in vivo* disease phenotype in mice. To overcome this challenge, we successfully replaced MrgprB2 with functional human MRGPRX2 in mouse BMMCs. Furthermore, their engraftment into MC-deficient mice results in normal migration and differentiation into connective tissue MCs with retention of their functional properties. Thus, our ability to stably express functional MRGPRX2 in mouse BMMCs and to demonstrate normal tissue distribution following their engraftment into MC-deficient mice provides unique opportunities to investigate MRGPRX2 functions *ex vivo* and disease phenotype *in vivo*.

Mouse BMMCs can be cultured in large numbers and are commonly used to study IgE-mediated responses *in vitro*. However, BMMCs do not express MrgprB2, and transient expression of MRGPRX2 has been used to show that they can be used to study human receptor function *in vitro* (61). Unlike BMMCs, mouse CTMCs, such as those found in the skin, peritoneum and gut, express MrgprB2 (3, 16). As the goals of the present study were to generate large numbers of BMMCs expressing MRGPRX2 for functional studies *ex vivo* and to engraft them into MC-deficient mice for tissue distribution studies, it was critical to utilize MrgprB2^–/–^ BM cells for retroviral transduction and their differentiation into BMMCs. We found that stable expression of MRGPRX2 in BMMCs had no effect on their phenotypic characteristics as they stained blue with alcian/safranin. Furthermore, MRGPRX2 expression did not have any effect on FcϵRI and c-Kit expression and they responded normally to antigen/IgE mediated MC degranulation. While all MRGPRX2 agonists use G proteins for Ca^2+^ mobilization and degranulation, others, such as SP, also promote β-arrestin-mediated receptor internalization, but LL-37 does not (24, 58). We therefore utilized SP and LL-37 as agonists to determine if they utilize the same signaling in transduced BMMCs as those reported in human MCs. Indeed, we found both agonists caused Ca^2+^ mobilization, degranulation and TNF-α production in BMMCs expressing MRGPRX2. By contrast, SP induced MRGPRX2 internalization, but LL-37 did not. Given that large numbers of BMMCs expressing MRGPRX2 can be generated following retroviral transduction of BM cells and culturing these cells in the presence of mIL-3, these cells can be used to study MRGPRX2 signaling in MCs.

Engraftment of BMMCs from genetically modified mice into MC-deficient mice, also known as the “MC knock-in” procedure, has been used extensively to study the effects of genetic modification of MCs on a variety of disease phenotypes. Although retrovirus-mediated transduction has been used to study how modification of FcϵRI and its signaling regulates functions of mouse BMMCs *ex vivo* (36, 37), to the best of our knowledge, these cells have not been engrafted into MC-deficient mice for *in vivo* studies. This study provides the first demonstration that engraftment of MRGPRX2-BMMCs into W^sh^/W^sh^ mice results in the normal tissue distribution of MCs, which retain the functional receptor expression. It is noteworthy that tissue distribution of engrafted BMMCs into MC-deficient mice is dependent on the route of administration. Thus, if BMMCs are injected into the skin, they do not migrate to other anatomical sites. By contrast, if BMMCs are administered intraperitoneally, they differentiate into CTMCs at this site and migrate to the gut. Furthermore, if BMMCs are administered intravenously, they migrate to additional sites, including the lung. For the present study, we utilized the peritoneal route of BMMCs engraftment into W^sh^/W^sh^ mice. We found that intraperitoneal administration of BMMCs resulted in their differentiation into CTMCs, as demonstrated by their staining properties with alcian/safranin. Furthermore, these MCs retained the expression of MRGPRX2, which was functional, as demonstrated by the ability of SP and C48/80 to induce degranulation. We have shown that engrafted MRGPRX2-BMMCs migrate to the small intestine and colon like engrafted control-BMMCs and retain receptor expression. For the present study, we used kit mutant W^sh^/W^sh^ mice for engraftment studies, but these mice have certain abnormalities independent of MCs (62). Depending on the disease model used, utilization of other MC-deficient mice that do not involve c-kit mutation should be considered.

Recent cryogenic electron microscopy studies revealed that MRGPRX2 has two shallow binding pockets with acidic, negatively charged residues within the receptor’s transmembrane domain that facilitate MRGPRX2 binding by its cationic ligands (34, 63). Furthermore, single-nucleotide polymorphisms (SNPs) on both the ligand and G-protein interfaces of MRGPRX2 have been identified (31, 32, 34, 57). Our lab has successfully characterized some of these gain and loss-of-function SNPs utilizing transfected RBL-2H3 cells *in vitro* (31, 32). Since CRISPR/Cas9-mediated gene targeting approach has been successfully utilized to replace endogenous MrgprB2 with MRGPRX2 (28, 29), generating mice expressing MRGPRX2 SNPs to determine receptor function *in vivo* should be possible. Humanized mice developed through human hematopoietic stem cells (hHSCs) engraftment into lethally irradiated immune-deficient mice have been used to study the role of human MCs on cutaneous drug reactions and IgE-mediated systemic anaphylaxis *in vivo* (64–67). It could also be possible to use CRISPR/Cas9 to introduce MRGPRX2 SNPs into hHSCs before their engraftment into immune-deficient mice. However, unlike the procedure developed in this study, neither of these techniques would be a cost and time effective approach to screen a large number of MRGPRX2 variants for *ex vivo* and *in vivo* studies. Thus, the protocol developed in this study should allow for generating BMMCs expressing MRGPRX2 and its variants on a large scale, to study how naturally occurring SNP regulate MC function *ex vivo* and disease phenotype *in vivo* and thus provide new information that is translationally relevant.

It is generally accepted that MRGRPX2/B2 is known to contribute to drug-induced pseudoallergy and cutaneous disorders. However, recent studies have shown that MrgprB2 also contributes to IgE-mediated lung inflammation, systemic anaphylaxis and ulcerative colitis (17–19, 68). These findings are consistent with the report that 10-20% of human lung and colonic MCs express MRGPRX2 (69). However, it should be possible to use retrovirus to express MRGPRX2 variants into MrgprB2^–/–^ BM cells and to differentiate them *ex vivo* for functional studies. Also, engraftment of MRGPRX2 variants into MC-deficient mice could provide a novel approach to determine the impact of receptor modification on both IgE and non-IgE-mediated disorders. In addition, G protein-coupled receptor kinase 2 (GRK2) and the adapter protein β-arrestin2 have been shown to regulate MRGPRX2-mediated responses *in vitro*, also MrgprB2-mediated rosacea, IgE-mediated allergic lung inflammation and systemic anaphylaxis *in vivo* (70–75). Thus, it should be possible to express MRGPRX2 in BM cells from mice deficient in GRK2 and β-arrestin2 and to differentiate them into BMMCs for signaling studies *ex vivo* and to determine how they modulate MRGPRX2-mediated IgE and non-IgE-mediated disorders following their engraftment into MC-deficient mice (6, 17, 18).

In conclusion, our detailed protocol represents a valuable platform for expressing MRGPRX2 or its SNPs in BMMCs, engrafting them via different routes, and utilizing them to study MRGPRX2 modulation, downstream signaling, and modification in IgE-dependent and independent MC-mediated disorders *in vivo*.

## Data Availability

The raw data supporting the conclusions of this article will be made available by the authors, without undue reservation.
